# Hazards of Malaysia’s Favorite Sport: A Case Series of Badminton-Related Ocular Injuries at a Single Tertiary Center

**DOI:** 10.7759/cureus.101594

**Published:** 2026-01-15

**Authors:** Fatin Nabila Mat Nawi, Mae-Lynn Catherine Bastion

**Affiliations:** 1 Ophthalmology, Hospital Canselor Tuanku Muhriz, Kuala Lumpur, MYS; 2 Ophthalmology, Faculty of Medicine, Universiti Kebangsaan Malaysia, Kuala Lumpur, MYS

**Keywords:** badminton-related ocular injury, protective eyewear, shuttlecock injury, sports-related eye injury, traumatic hyphaema, traumatic uveitis

## Abstract

Badminton is a popular recreational and competitive sport in Southeast Asia but carries a considerable risk of ocular injury due to high-velocity shuttlecock and racket impacts, as well as spectacle fragmentation. This series reported seven cases of badminton-related ocular trauma. Seven players in this series were predominantly young adults, with men representing the majority of cases. Injuries resulted primarily from shuttlecock strikes, while one patient sustained a penetrating wound from shattered spectacles. Clinical presentations ranged from subconjunctival hemorrhage, traumatic uveitis, traumatic hyphema, and commotio retinae to open-globe injury with corneoscleral laceration. Most cases were managed medically with topical therapy, while surgical intervention was required only for the open-globe injury. Visual outcomes correlated with injury severity, with penetrating trauma resulting in a poorer prognosis. This series highlights badminton playing as a significant source of potentially preventable ocular morbidity and emphasizes the importance of protective eyewear, particularly among spectacle users and young players. Increased awareness and enforcement of eye protection standards may reduce the risk of visual impairment associated with this widely played sport.

## Introduction

Sport-related ocular trauma remains a major cause of preventable visual morbidity worldwide. Previous reports indicate that a substantial proportion of ocular injuries are related to sporting activities, accounting for a significant share of hospital admissions for eye trauma [1,2]. Badminton, one of the most widely played sports in Southeast Asia, has been identified as a notable contributor to ocular trauma apart from musculoskeletal injuries [3,4]. In Malaysia, badminton accounts for nearly two-thirds of sport-related eye trauma cases [3], while a United Kingdom series attributed 14.3% of sport-associated eye injuries to badminton [5].

Ocular injury in badminton typically results from either a high-speed shuttlecock strike or impact from a racket. The shuttlecock’s small, rigid base can produce significant blunt force, generating abrupt anterior-posterior compression of the globe and equatorial expansion. This mechanism may precipitate acute conditions such as hyphema, traumatic cataract, cyclodialysis, iridodialysis, sphincter tears, lens subluxation, vitreous hemorrhage, and Berlin edema [4-6]. Delayed sequelae including cystoid macular edema, retinal detachment, traumatic optic neuropathy, macular pigmentary changes, and angle recession glaucoma have also been reported [4,6]. In addition, shattering of prescription spectacles from direct impact may result in penetrating injuries and devastating visual consequences.

## Materials and methods

This retrospective case series was conducted at a single tertiary ophthalmology referral center in Universiti Kebangsaan Malaysia Medical Centre (Hospital Canselor Tuanku Muhriz), Kuala Lumpur, Malaysia. The case records of patients who presented with badminton-related ocular trauma between March 2021 and November 2025 were retrospectively reviewed.

Inclusion and exclusion criteria

Patients were included if they sustained ocular injuries directly related to badminton play and were evaluated by the ophthalmology service during the study period. Seventeen patients presented with badminton-related ocular injuries, but only seven cases with available initial presentation photographs were included. Ten patients were excluded as initial presentation images were unavailable.

Data collection

Cases were initially identified through an ophthalmology trainee's on-call handover communication platform. Patients’ records were then traced from the record office. Clinical data collected include patient demographics (age, gender), mechanism of injury, clinical examination findings, visual acuity (VA) at presentation, intraocular pressure (IOP), clinical images, initial treatment and management strategies, and short-term follow-up outcomes.

Outcome measures

The primary outcome was the spectrum and severity of ocular injuries resulting from badminton-related trauma at presentation. Secondary outcomes included the initial management approach (medical and/or surgical) and monitoring plan.

## Results

Table 1 summarizes all seven cases described in this series.

**Table 1:** Summary of 7 reported cases. VA: visual acuity; RAPD: relative afferent pupillary defect; AC: anterior chamber; RBCs: red blood cells; IOP: intraocular pressure; CF: counting fingers vision; PSCC: posterior subcapsular cataract

Case	Age/gender	Mechanism of injury	Anterior segment of the affected eye	Posterior segment of the affected eye	Treatment
1	31 y/o male	Blunt shuttlecock injury to left eye	VA 6/12, no RAPD, conjunctival injection, subconjunctival hemorrhage, AC deep with RBCs, sluggish pupil, splinter tear, IOP 28 mmHg	Commotio retinae	Prednisolone acetate q2h, timolol, atropine TID
2	16 y/o male	Spectacles shattered and hit right eye	VA CF, multiple globe lacerations, flat AC with iris prolapse, oval pupil, iridodialysis, PSCC	No B-scan, no IOP recorded	Urgent globe repair, oral ciprofloxacin, topical Maxidex, ciprofloxacin, atropine
3	40 y/o female	Shuttlecock struck the glasses on the right eye	VA 6/15, no RAPD, superficial lid lacerations, conjunctival abrasion, mild subconjunctival hemorrhage, clear cornea, deep AC	Normal fundus	Chloramphenicol ointment and drops, artificial tears
4	9 y/o boy	Shuttlecock injury to left eye	VA 6/12, irregular pupil, corneal epithelial defect, hyphema, AC cells, elevated IOP of 23.6	Berlin edema, commotio retinae	Bed rest, topical dexamethasone, Ciloxan, atropine, timolol, oral paracetamol
5	17 y/o male	Shuttlecock injury to right eye	VA 6/6, mild conjunctival injection, hyphema < 1 mm, IOP 16 mmHg, clear lens	Normal fundus	Topical dexamethasone q2h, atropine, head elevation
6	37 y/o male	Partner's racket hit left eye	VA 6/36, dilated sluggish pupil, periorbital hematoma, AC cells 3-4+, clear lens, IOP 9 mmHg	Fundus not visible in left eye	Topical dexamethasone q2h, atropine TID, oral paracetamol
7	62 y/o male	Shuttlecock injury to right eye	VA hand movements, hyphema 1.6 mm, 4+ AC cells, IOP of 25 mmHg, limited iris view	B-scan; flat retina	Topical dexamethasone q2h, cyclopentolate TID, bed rest

**Figure 1:**
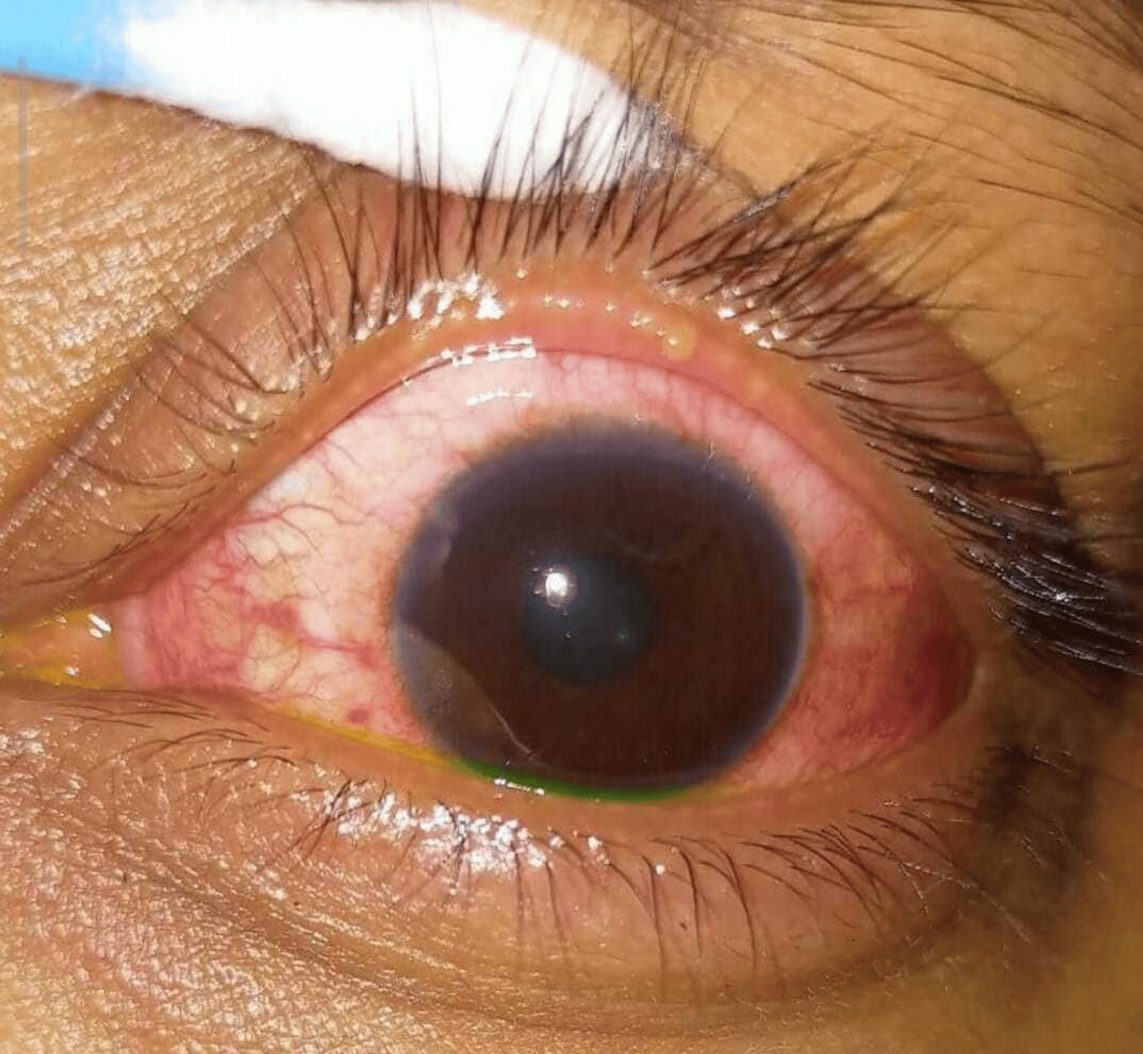
Case 1 showing the left eye with conjunctiva injection, small subconjunctival hemorrhage nasally.

Case 1 involved a 31-year-old man who sustained a high-velocity blunt trauma to the left eye caused by a shuttlecock. He presented with a headache and ocular redness. On examination, his left VA was 6/12 with no relative afferent pupillary defect (RAPD). The left eye showed conjunctival injection and a small subconjunctival hemorrhage nasally (Figure 1). The anterior chamber (AC) was deep with 2-3+ red blood cells. The pupil was sluggish, measuring 4 mm, and a splinter tear was observed at the 9 o’clock position. Fundus examination was limited, but inferior commotio retinae was noted. IOP was elevated at 28 mmHg. The patient was started on prednisolone acetate every two hours, timolol twice daily, and atropine three times daily. Improvement in inflammation and IOP control was anticipated; however, follow-up data were not available.

**Figure 2:**
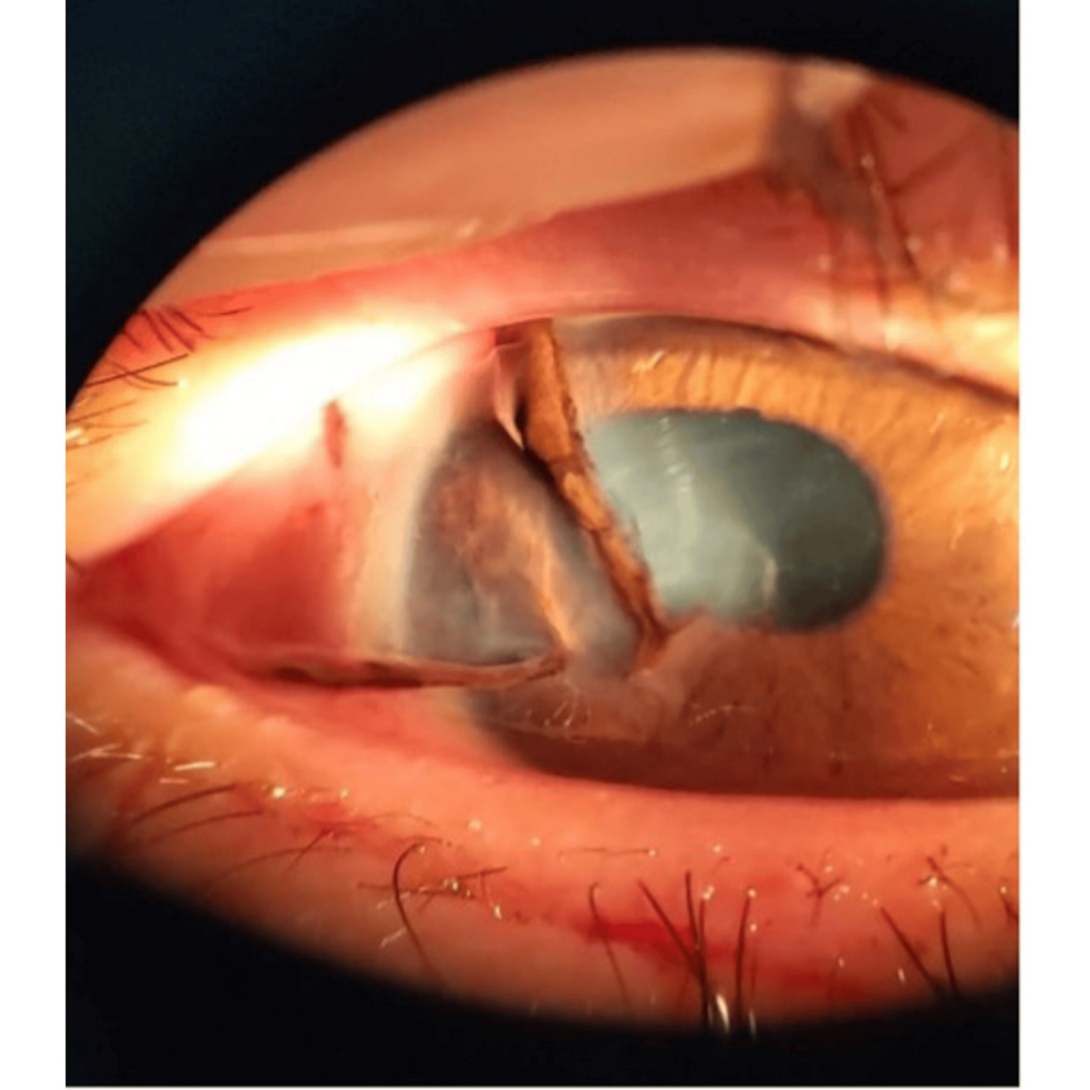
Case 2 showing corneoscleral laceration wounds at 8 and 10 o’clock measuring 10 and 8 mm, respectively, with prolapsed iris through the wounds. Another scleral wound at the superotemporal measuring 3 mm. The anterior chamber was flat. No hyphema seen. Iridodialysis was present at 8-9:30 o’clock.

Case 2 illustrates a more severe injury involving globe lacerations and surgical intervention. A 16-year-old male patient suffered severe right eye trauma when his glass spectacle lens shattered upon impact. Presenting VA was counting fingers. Examination revealed conjunctival injection and multiple globe lacerations, including corneoscleral wounds measuring 10 mm at 8 o’clock, 8 mm at 10 o’clock, and a 3 mm superotemporal scleral laceration (Figure 2). The AC was flat with fibrin, and the pupil was oval with iridodialysis between 8 and 9:30 o’clock and at 10 o’clock. Posterior segment visualization was not possible; no B-scan was performed, and IOP was not recorded. Urgent surgical globe repair was performed to restore globe integrity, with a guarded prognosis. By postoperative day 6, VA improved to 6/60 and IOP was 14 mmHg. Commotio retinae was noted outside the superior and inferior arcades. Treatment included oral ciprofloxacin and topical dexamethasone and ciprofloxacin every two hours, with atropine once daily. Three weeks post-trauma, a grade 2+ posterior subcapsular cataract (PSCC) developed. At one month post-trauma, he underwent corneal suture removal and resuturing, complicated by wound leaks requiring corneal glue application. Nearing six months post injury, corneal glue removal, synechiolysis, and lens aspiration were performed, resulting in aphakia. Final VA was 6/60, improving to 6/18 with pinhole, and near vision was N24.

**Figure 3:**
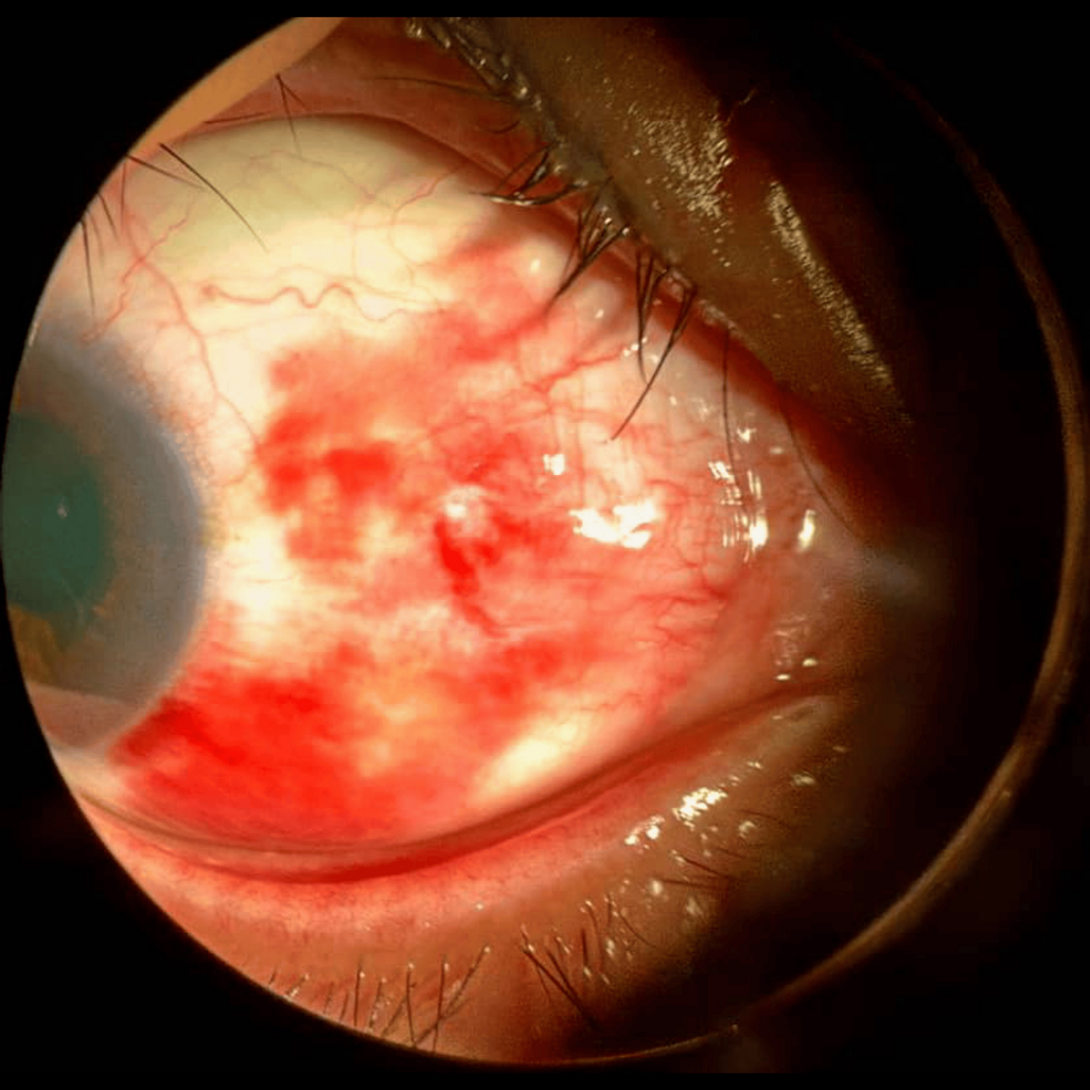
Case 3 showing the right eye with subconjunctival hemorrhage nasally attributed to a conjunctival defect.

In contrast, case 3 involved a less severe injury with superficial trauma and a favorable prognosis. A 40-year-old female patient with no known medical illnesses presented with redness and tearing of the right eye following trauma sustained during a badminton game, where a shuttlecock struck her glasses, causing them to chip and subsequently impact her right eye. She denied blurred vision, floaters, or flashes. Unaided VA was 6/15 in the right eye and 6/24 in the left, improving to 6/9 with pinhole bilaterally. No RAPD was noted. Extraocular movements were full without pain or diplopia. The right eye showed superficial skin lacerations on the upper and lower eyelids, sparing the lid margin and lacrimal drainage system. Lid eversion and sweeping revealed no foreign bodies. Conjunctival abrasion with nasal subconjunctival hemorrhage was noted without chemosis (Figure 3). The cornea was clear. The AC was deep and quiet, pupil round and reactive, and lens clear. Right eye fundus examination showed a pink optic disc with a cup-to-disc ratio (CDR) of 0.3, a normal macula, and a flat retina. The left eye was unremarkable. The patient was treated with chloramphenicol ointment three times daily for periorbital wounds, chloramphenicol eye drops four times daily, and preservative-free artificial tears every two hours in the right eye. Follow-up data were not available.

**Figure 4:**
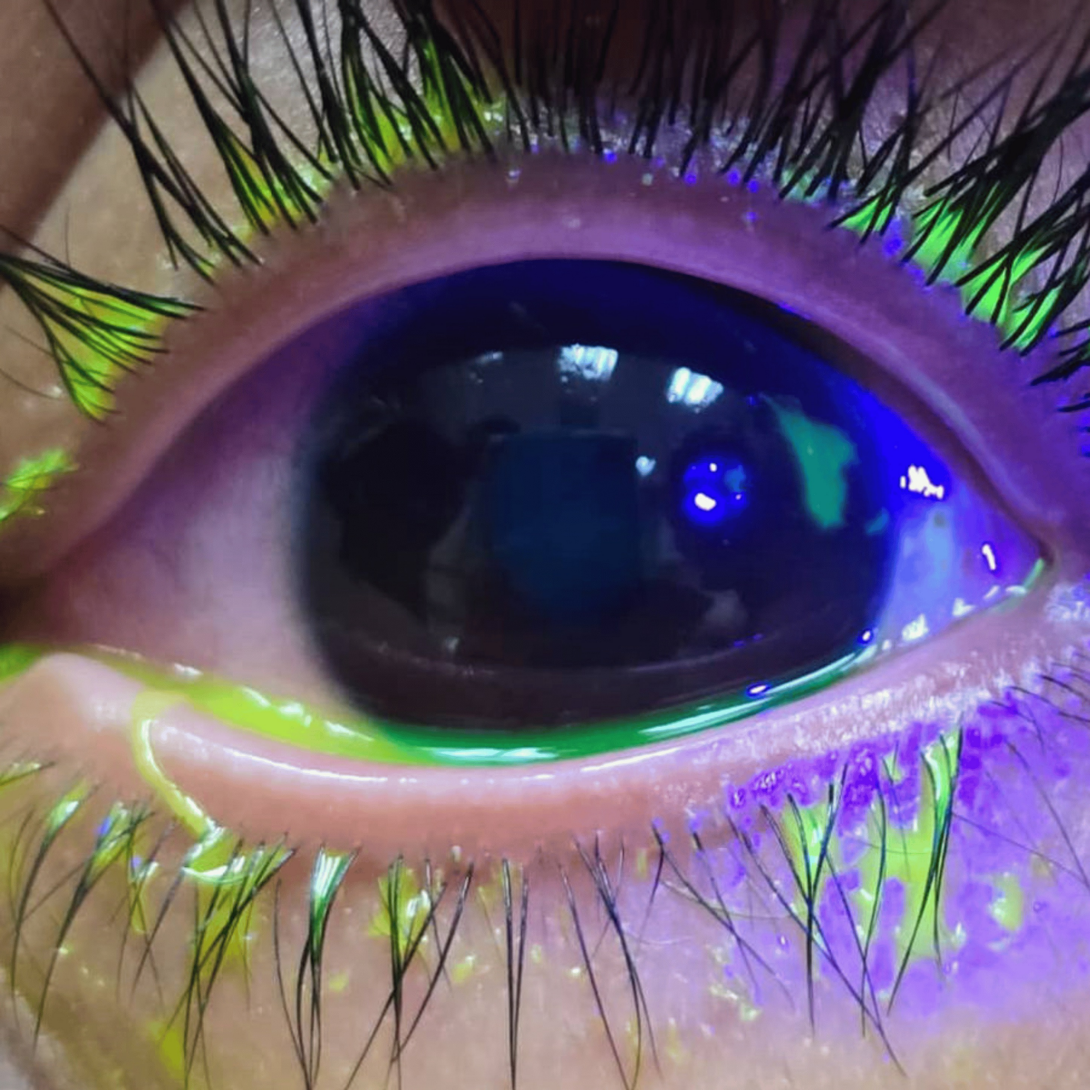
Case 4: corneal epithelial defect at periphery 2 o'clock.

**Figure 5:**
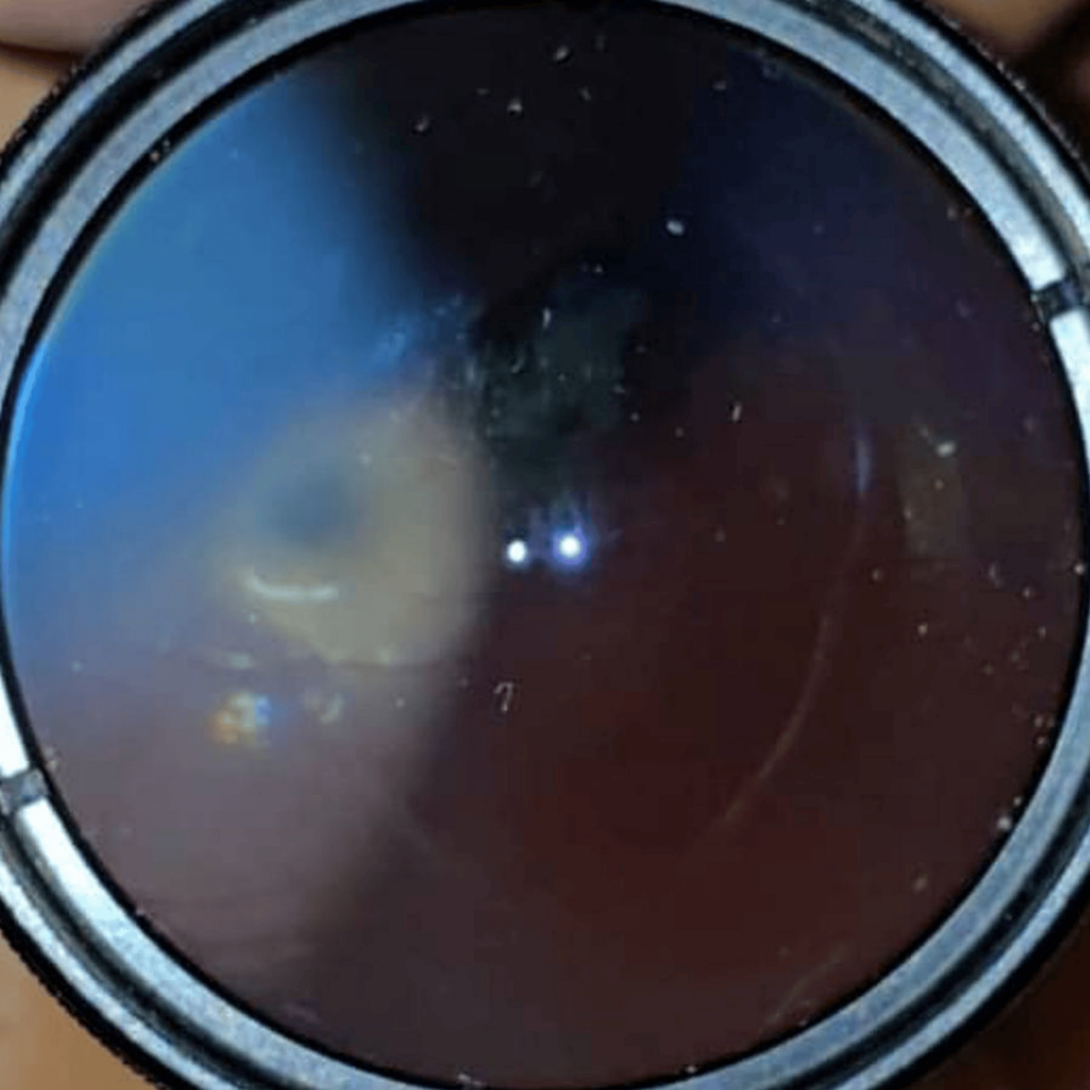
Case 4: left eye fundus showing commotio retinae measuring about 1.5 disc diameter size.

**Figure 6:**
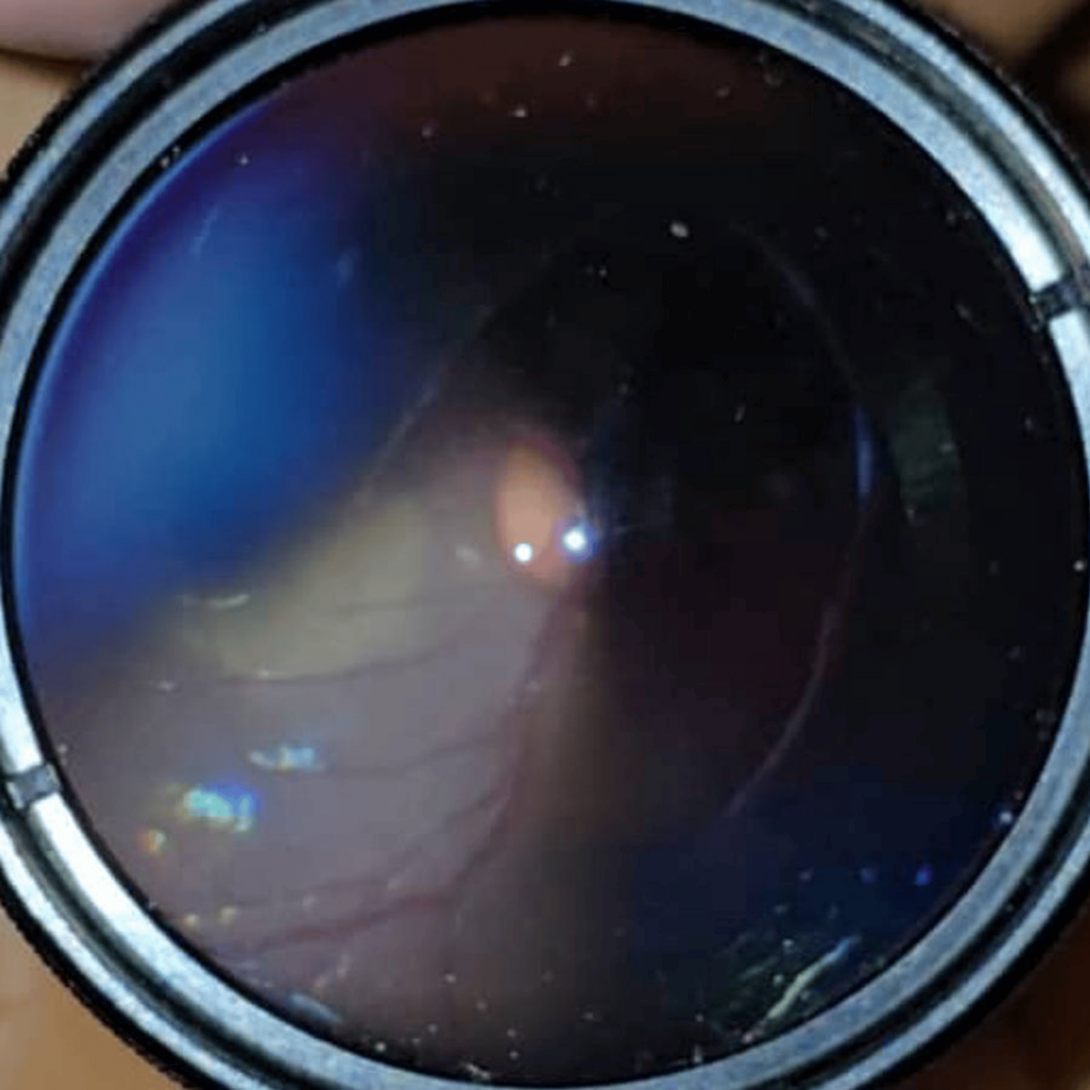
Case 4: left eye fundus examination using a binocular indirect ophthalmoscope showing a similar commotio retinae (Berlin edema) location in relation to the optic disc.

Case 4 demonstrates ocular trauma in a pediatric patient with complex anterior and posterior segment involvement. A nine-year-old boy with no known medical illness presented following an ocular trauma sustained during a badminton game. Another 11-year-old child accidentally struck the patient’s left eye with a shuttlecock; however, the exact force and velocity of impact were uncertain. Post-injury, the patient experienced eye redness, eye pain, and blurred vision in the left eye, accompanied by a mild headache. There was no diplopia, metamorphopsia, head injury, vomiting, scotoma, or photopsia reported. On examination, RAPD was negative. The right pupil measured 3 mm, while the left pupil was irregularly shaped, measuring 4-5 mm. VA was 6/6 in the right eye and 6/12 in the left eye. Extraocular movements were full bilaterally without diplopia, though the patient reported pain on eye movement correlating with a corneal epithelial defect. Left eyelids showed mild erythema without swelling. Conjunctival injection was present without chemosis or conjunctival defects. A small corneal epithelial defect measuring approximately 2 mm vertically by 1 mm horizontally was noted near the 2 o’clock limbal region (Figure 4). The AC was deep with 4+ cells and a 1 mm level of hyphema. The left pupil was eccentric at 4-5 mm. The lens was clear with no phacodonesis. IOP measured by I-care was 17.1 mmHg in the right eye and elevated at 23.6 mmHg in the left eye. Fundus examination revealed a normal right eye. The left eye showed a pink optic disc with a CDR of 0.3. There was macular Berlin edema approximately 1.5 times the optic disc size (Figures 5, 6) and extensive commotio retinae over the nasal retina. No vitreous hemorrhage, retinal tears, or breaks were observed. The diagnosis included left eye traumatic hyphema grade I with secondary ocular hypertension, commotio retinae with Berlin edema, traumatic mydriasis, and corneal abrasion. Management comprised strict bed rest with head elevation at 45 degrees, avoidance of heavy lifting, and topical medications including dexamethasone every two hours, ciprofloxacin every four hours, atropine 1% once daily, and timolol twice daily in the left eye, along with oral paracetamol 300 mg four times daily for pain control. The patient subsequently recovered.

**Figure 7:**
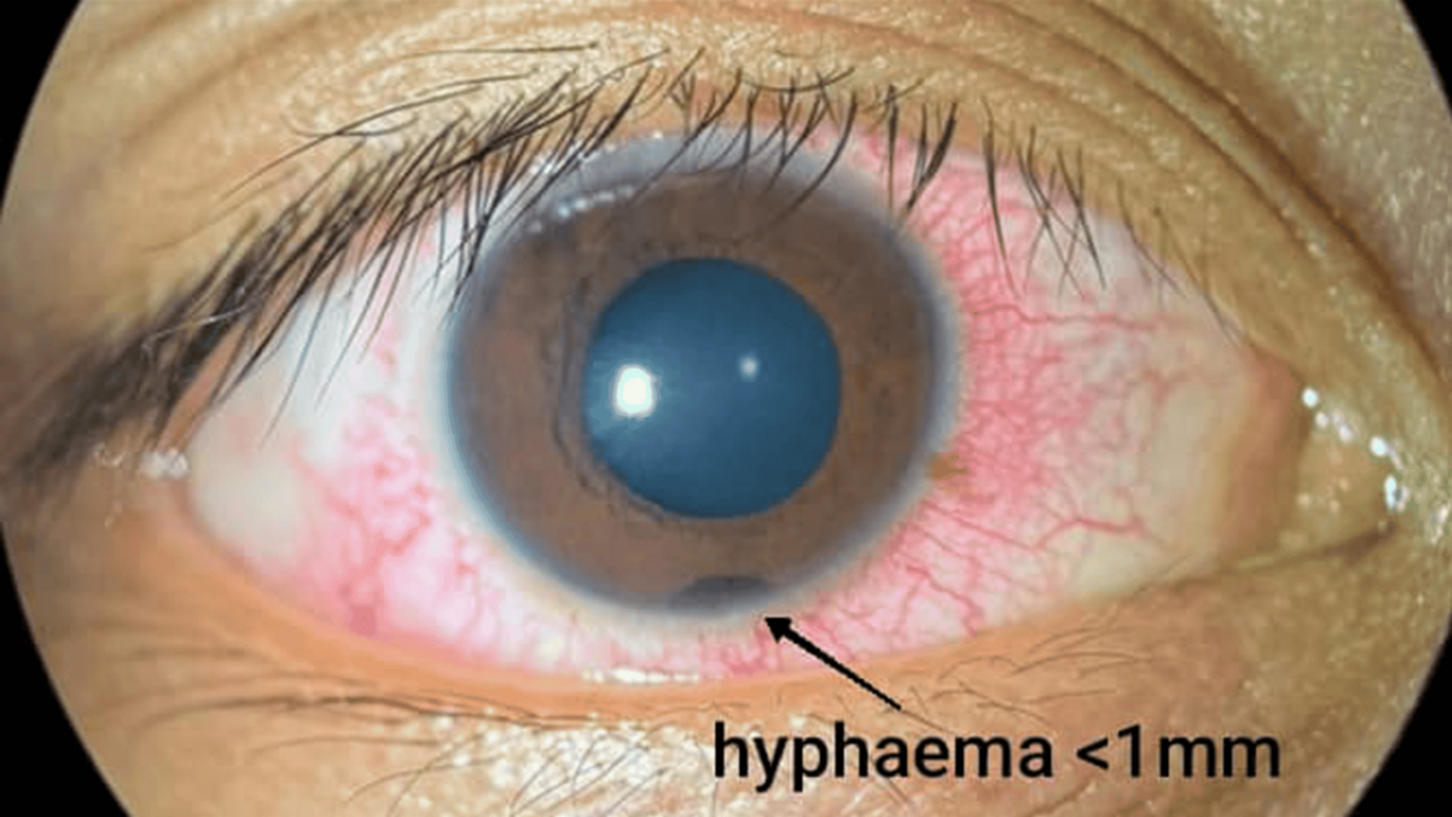
Case 5 showing mildly injected conjunctiva and hyphema < 1 mm.

Case 5 described a 17-year-old male patient with no known medical illnesses who presented following an alleged shuttlecock injury to the right eye. The patient reported that the shuttlecock lightly struck the area above his right eye; it was unclear whether his eye was closed at the time of injury. Post-trauma, he experienced right eye pain rated 4 out of 10 and eye redness. He denied any visual disturbances, flashes, or floaters. He was not using any corrective lenses. On examination, VA was 6/6 bilaterally with near vision N5. There was no RAPD. Pupils were equal, round, and reactive at 3 mm in both eyes. The right eyelid showed no swelling, erythema, or hematoma. The conjunctiva was mildly injected without subconjunctival hemorrhage (Figure 7). The cornea was clear. AC was deep with the presence of hyphema measuring less than 1 mm and 4+ red blood cells (Figure 7). IOP measured 16 mmHg. The lens was clear with no evidence of phacodonesis. Fundus examination of the right eye revealed a pink optic disc with a CDR of 0.4, a healthy macula with a good foveal reflex, and a flat retina without tears, commotio retinae, or hemorrhages. The left eye anterior segment and fundus were unremarkable. The patient was managed with topical dexamethasone every two hours and atropine eye drops in the right eye. He was advised to maintain a head-elevated position and avoid strenuous activities and was put under closed follow-up.

**Figure 8:**
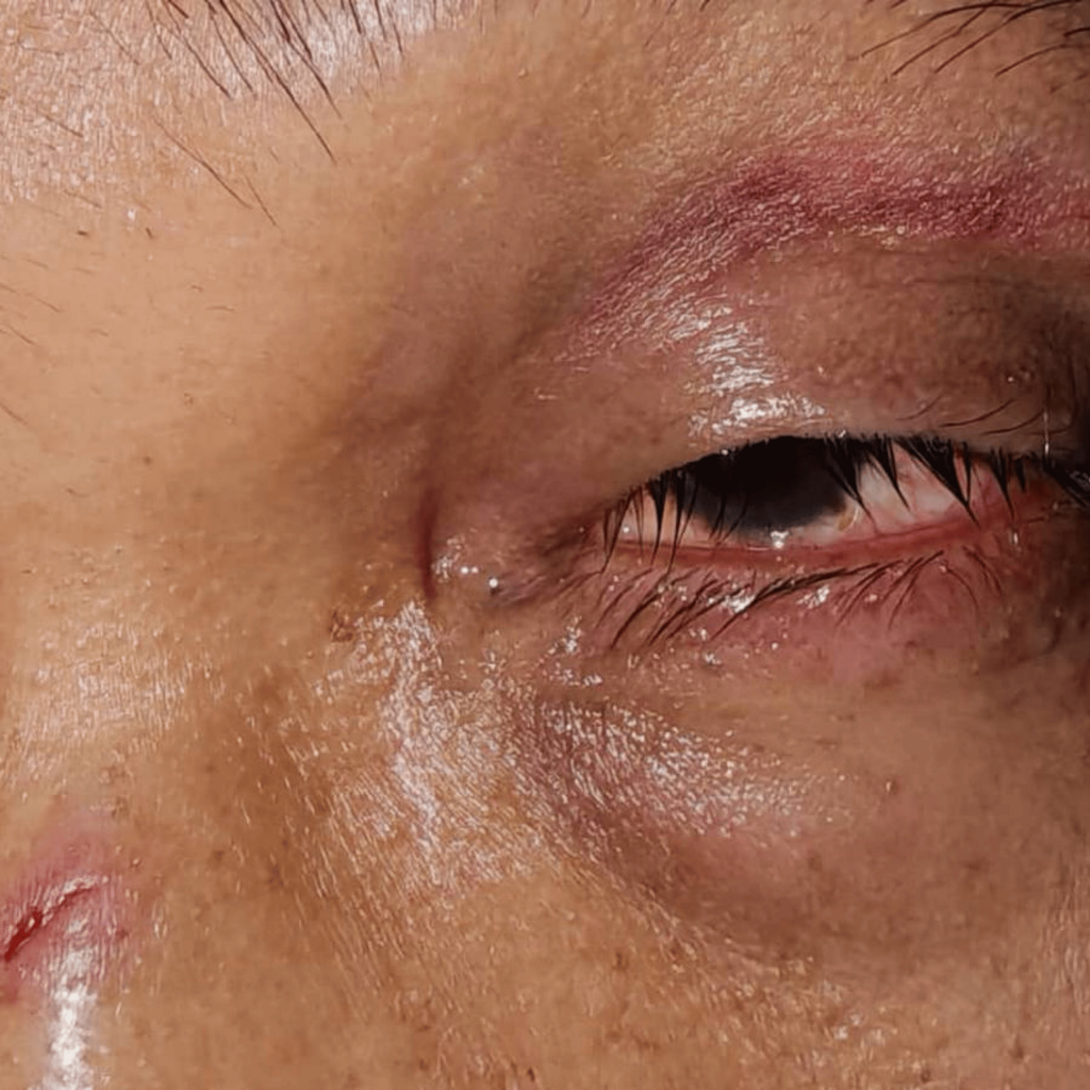
Case 6 showing mild periorbital hematoma with mild conjunctival injection and small superficial laceration wound at the nasal bridge.

Case 6 highlights a case of a 37-year-old male patient with underlying hepatitis B who presented with left eye pain following a high-velocity injury sustained while playing badminton. His playing partner’s racket accidentally struck his left eye. He experienced significant localized pain and generalized blurred vision. Initial pain was rated 8/10 but gradually improved. The patient was not wearing any corrective lenses at the time of injury. There was no bleeding or discharge. VA was 6/9 (pinhole 6/6) in the right eye and 6/36 (pinhole 6/24) in the left eye. No RAPD was detected. The right pupil was 2 mm, round, and reactive; the affected left pupil was dilated to 4 mm and sluggishly reactive. A small superficial laceration was noted over the nasal bridge (Figure 8). The left eye had a mild periorbital hematoma without wounds or step deformity (Figure 8). The conjunctiva was mildly injected; the cornea was clear. The AC was deep with 3-4+ cells; no hyphema was present. The lens was clear. IOP was 9 mmHg in the left eye. Fundus examination of the left eye was not possible. The right eye anterior segment was normal with an IOP of 10 mmHg, a pink optic disc with a CDR of 0.3, a normal macula, and a flat retina. The clinical impression was left eye traumatic mydriasis with uveitis. Treatment included topical dexamethasone every two hours and atropine eye drops three times daily in the left eye, and oral paracetamol 1 g three times daily for pain. A close follow-up was scheduled to monitor the AC reaction and treatment response.

**Figure 9:**
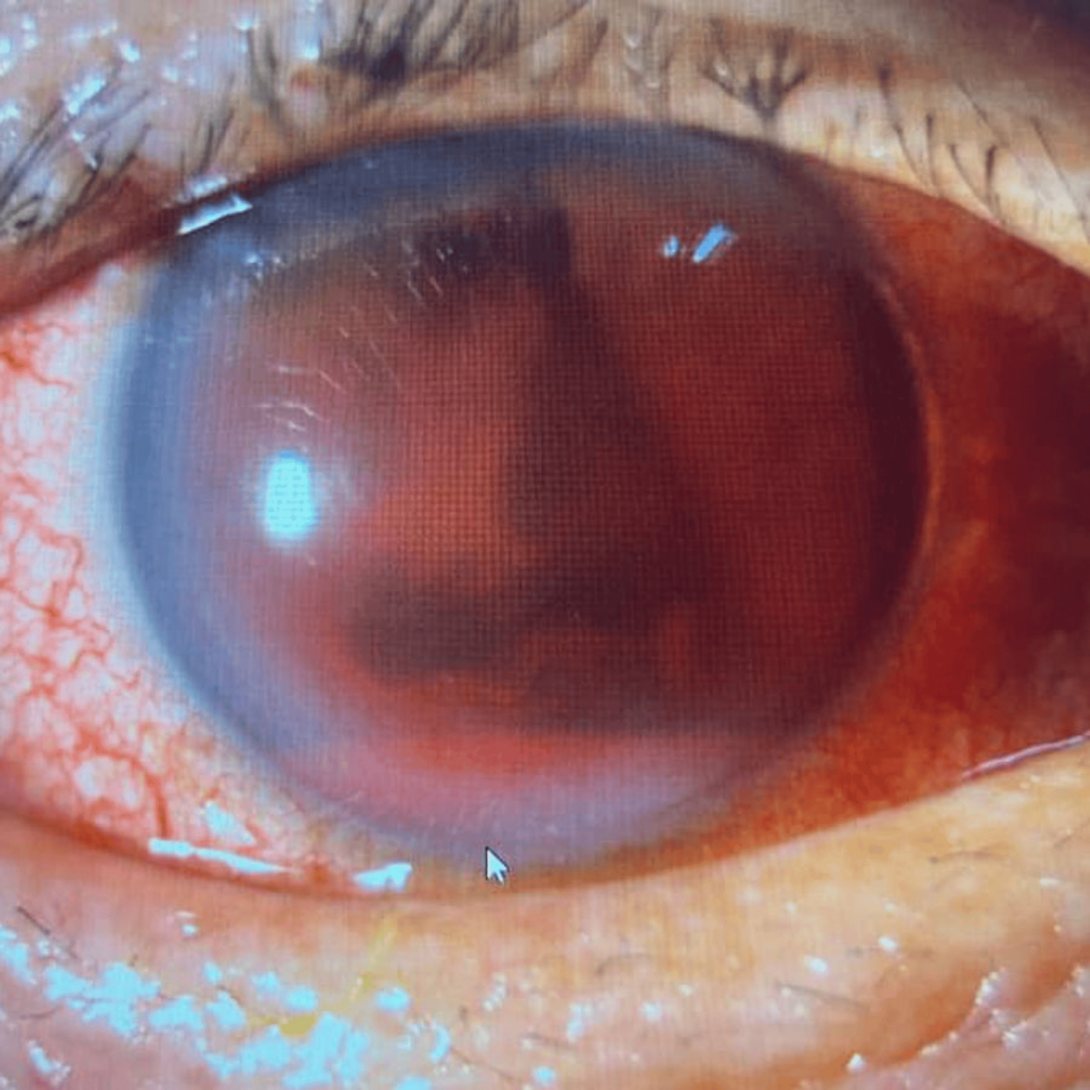
Case 7 showing hyphema level and bleeding obscuring the visual axis.

**Figure 10:**
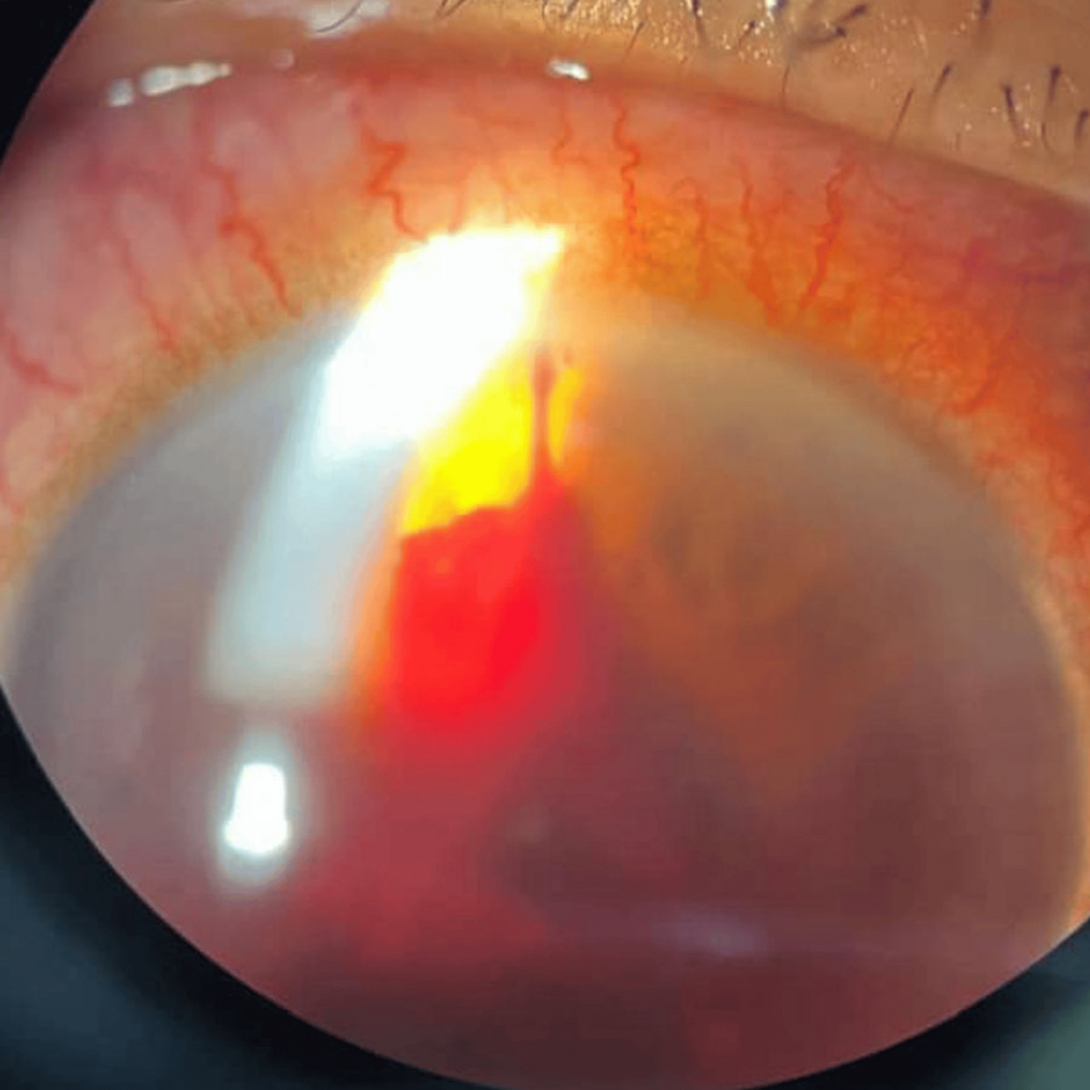
Case 7 showing a vertical stream of blood from the iris at 12 o'clock.

Finally, case 7 describes an elderly patient with significant hyphema and elevated IOP requiring close monitoring. A 62-year-old man with dyslipidemia and insomnia under psychiatric care presented after an alleged shuttlecock injury to the right eye. The injury occurred when his opponent returned the shuttlecock via an overnet tap shot from approximately two arm’s lengths away. The patient was unable to fully close his right eye, resulting in direct impact on the ocular region. Post-trauma, he experienced transient blurred vision in the right eye, which resolved spontaneously within 1-2 hours, with vision returning to baseline. He denied floaters, flashes, or visual field defects on the day of trauma. On the morning of presentation, the patient reported a sudden onset of generalized "obscured" vision in the right eye upon waking, without preceding flashes, floaters, or curtain-like vision loss. He denied eye pain. Eye redness was persistent. Examination revealed no RAPD. VA was hand movements in the right eye and 6/9 near N6 in the left eye. The right eyelids were normal. The conjunctiva was white, and the cornea was clear without epithelial defects or lacerations. The AC showed a hyphema measuring 1.6 mm inferiorly with 4+ cells (Figure 9). A vertical stream of blood clots was observed from the iris at 12 o’clock, suspected as the bleeding source (Figure 10). Iris view was limited superiorly and nasally. IOP was 25 mmHg in the right eye. Fundus view was not possible. B-scan ultrasonography showed no vitreous opacities and a flat retina. The left eye was unremarkable with an IOP of 10 mmHg and normal fundus (pink optic disc, CDR 0.6, and flat retina). The clinical impression was right eye grade III traumatic hyphema secondary to shuttlecock injury. Treatment included topical dexamethasone every two hours and cyclopentolate three times daily in the right eye. The patient was advised complete bed rest with head elevation of at least 30 degrees and on close IOP monitoring. The patient is still under ophthalmology clinic follow-up.

In a nutshell, patients ranged from nine to 62 years old; six were male. Only one eye per patient was affected. Four injuries resulted from direct shuttlecock impact, two from spectacle fragmentation, and one from racket hit. Common presenting complaints included ocular pain, redness, and blurred vision. Most patients demonstrated a relatively good initial VA, while two had significantly reduced vision (counting fingers and hand movement). Corneoscleral laceration and hyphema accounted for severe visual impairment. Traumatic uveitis was observed in the majority of cases. Surgical intervention was required only for the penetrating injury case, while others were managed with topical eyedrops, mainly topical steroids.

## Discussion

Badminton players are susceptible to multiple types of ocular trauma, with an estimated injury incidence rate among elite players at 5.04 per 1,000 playing hours [7]. Consistent with existing literature, males are more frequently affected [8], and the majority of patients in our study were male. Injuries may result from a shuttlecock smash, player movement within doubles play, or racket contact. The shuttlecock remains the most common causative agent [9,10], with its eyeball-sized rigid base capable of generating significant ocular damage [9]. The sharp, feathery edges of the shuttlecock may contribute to penetrating ocular trauma.

Shattered spectacles represent another important injury mechanism, and penetrating ocular injury resulting from these shattered glasses is associated with poorer visual prognosis [6]; therefore, protective alternatives such as polycarbonate lenses or sport-rated eyewear are recommended. Previous studies reported up to 29.4% of ocular trauma caused by racket strikes [4], capable of breaking spectacles and inflicting substantial injury. Only one racket injury case was described in this series.

In this study, reported ocular injuries involved are traumatic uveitis, commotio retinae, traumatic hyphema, globe injury, traumatic cataract, periorbital hematoma, and subconjunctival hemorrhage. As per other reported series, traumatic uveitis was frequently observed in shuttlecock-related injuries and has been attributed to the high-velocity impact on the uveal tissue [6,9].

Commotio retinae, usually transient, results from photoreceptor disruption and fluid accumulation secondary to countercoup injury [11]. Although typically self-limited, vision may be affected when the macula is involved. Two out of three cases with commotio retinae had initial good vision of 6/12. One clinical audit, however, revealed poorer final visual outcomes in commotio retinae cases, perhaps due to the more severe disruption of the outer segment of the photoreceptor causing functional damage and other concurrent injuries [9]. No vitreous hemorrhage was detected in this cohort, which may have otherwise obscured retinal assessment of commotio retinae and also contributed to poorer presenting VA.

Traumatic hyphema is one of the most reported ocular findings in shuttlecock-related trauma, with an incidence ranging from 66.7% to 96% [4,10,12], and contributes significantly to angle recession risk [13]. Hyphema occurs due to shearing or laceration of intraocular vessels, especially the iris vessels, by the force of the shuttlecock or racket. Injuries to the iris, such as sphincteric tears, iridodialysis, and cyclodialysis cleft, are also responsible for hyphema [13]. One out of three hyphema cases in this series has poor hand movement vision.

In an Australian study conducted on 12 patients with blunt ocular trauma while playing badminton, the incidence of angle recession and angle recession glaucoma was 58.3% and 41.6%, respectively [12]. Another study reported angle recession at 20%-94% post ocular injury and 5%-20% progressing into angle recession glaucoma eventually [14]. Angle recession occurred due to the shuttlecock impacting the eye with great force, causing separation of the longitudinal and circular fibers of the ciliary muscle [13]. Progression to angle recession glaucoma is more likely if more than 180 degrees of the angle is involved [14]. Three cases have elevated IOP of 23-28 mmHg, possibly due to the presence of AC inflammation and hyphema; however, the development of angle recession should also be assessed later.

The only penetrating injury case in our study involved a teenager with shattered glasses sustaining corneascleral laceration wounds. It is worth noting that even without broken spectacles, globe rupture can occur owing to the biomechanical susceptibility of the eye to deformation under force [15].

Badminton-related eye injuries from other literature include lens subluxation (27%) [4,6], secondary glaucoma [6], retinal detachment (13.7%) [4,6], vitreous hemorrhage (5.9%) [4,6], retinal hole/tear [9,10], and choroidal rupture [4]. The usage of protective eyewear, even during recreational sports, should be emphasized to prevent monocular blindness affecting job opportunities and quality of life. The lack of compulsory protective eyewear leaves amateur and professional players at risk [4].

Polycarbonate eyewear, despite cost and limitations in optical clarity, remains the most impact-resistant option [16]. Formal regulations have been adopted in some regions, such as mandatory protective eyewear for junior badminton players in Ontario [17] and eye protection for spectacle-wearing child athletes in New Jersey [18]. In Malaysia, despite badminton’s popularity, implementation remains advisory rather than enforceable.

This study is limited by its retrospective design, small sample size, and incomplete follow-up data, which constrain the ability to assess long-term visual prognosis and complications such as angle recession glaucoma. Nevertheless, these findings reinforce the significant ocular morbidity associated with badminton-related trauma and the necessity for increased awareness and preventive strategies within the sporting community. Another limitation is selection bias, as only cases with available initial clinical photographs were included, despite additional badminton-related ocular trauma cases occurring during the study period.

## Conclusions

Badminton poses a substantial yet under-recognized risk for ocular trauma, with the potential for irreversible visual consequences if injuries are severe or inadequately treated. The spectrum of injury ranges from mild anterior segment inflammation to severe penetrating trauma with poor visual prognosis. While many cases improve with medical therapy, penetrating injuries continue to demonstrate significantly poorer visual outcomes, highlighting the dangers associated with high-velocity shuttlecock impact, racket contact, and spectacle fragmentation. The rising prevalence of myopia may further heighten vulnerability among spectacle-wearing players. Although Malaysia has broad safe-sport frameworks, sport-specific regulations mandating eye protection for badminton are not clearly established; therefore, this case series highlights the need for greater awareness and education among players, coaches, and sporting communities regarding the mechanisms and preventable nature of badminton-related ocular trauma, particularly in young adults who represent the most active player demographic. Usage of polycarbonate protective sport eyewear represents an evidence-based measure with the potential to reduce the burden of avoidable visual impairment associated with this widely played sport.
